# Plasma bile acids in healthy green iguanas and iguanas with chronic liver diseases

**DOI:** 10.17221/10/2023-VETMED

**Published:** 2023-09-25

**Authors:** Zdenek Knotek, Zora Knotkova, Eva Cermakova, Gerry Maria Dorrestein, Kim Oliver Heckers, Dominik Komenda

**Affiliations:** ^1^Avian and Exotic Animal Clinic, Faculty of Veterinary Medicine, University of Veterinary Sciences Brno, Brno, Czech Republic; ^2^Diagnostic Laboratory NOIVBD, Vessem, The Netherlands; ^3^Laboklin GMBH, Bad Kissingen, Germany; ^4^Department of Imaging Methods, Faculty of Veterinary Medicine, Dog and Cat Clinic, University of Veterinary Sciences Brno, Brno, Czech Republic

**Keywords:** blood, hepatocellular carcinoma, liver adenoma, liver lipidosis, reptiles

## Abstract

The aim of the study was to establish reference values for plasma bile acid (BA) concentrations in a collection of healthy green iguanas and to compare the results with BA concentrations in iguana patients presented to the clinic with various types of chronic liver diseases, patients with other chronic diseases and healthy iguanas that were presented for routine or pre-surgical health check-up. The concentration of BA was determined using the enzymatic colorimetric method. Mean plasma bile acid concentration in 110 samples from healthy green iguanas fasted for 24 h was higher (15.89 ± 15.61 μmol/l) than plasma bile acid concentration in the same iguanas fasted for 48 h (9.56 ± 8.52 μmol/l) (*P* < 0.01). The 3α-hydroxy bile acid concentration was significantly altered in 9 patients suffering from chronic liver diseases (diagnosed by histology) (84.85 ± 22.29 μmol/l). BA concentration in one iguana with hepatocellular adenoma (13.0 μmol/l) was within the interval of BA in healthy iguanas. Mean plasma BA concentration in 10 green iguanas that were suffering from various types of chronic diseases, but without any hepatopathy was 7.85 ± 4.86 μmol/l. The mean plasma BA concentration in 18 samples from green iguanas presented to the clinic for routine health check-ups and 17 green iguana females with preovulatory follicle stasis (POFS) syndrome presented for ovariectomy was 11.95 ± 9.43 μmol/l and 12.97 ± 9.06 μmol/l, respectively. The data collected from this study suggest that plasma bile acids are significantly increased in green iguanas suffering from chronic liver diseases.

While acute liver diseases in reptiles may be associated with elevations of several enzyme activities including aspartate aminotransferase and lactate dehydrogenase, there is still a lack of information concerning the existence of a feasible method for monitoring chronic liver failure in reptiles (Campbell 2004; Divers 2020). Studies that have been performed to characterise the bile acids in reptiles demonstrate that a variety of different bile acids are produced, nevertheless, 3-α bile acids appear to be conserved amongst all reptile groups (Schaffner 1998; McBride et al. 2006; Divers 2019). As the clinical symptoms of liver failure in reptiles are nonspecific the attention is focused on methods of indirect diagnostics, such as diagnostic imaging, clinical haematology and plasma chemistry (Divers and Cooper 2000; Hernandez-Divers and Cooper 2006). Monitoring of bile acid concentrations (BA) in the peripheral blood of reptiles is still at the centre of attention because elevated values are supposed to be present in patients suffering from chronic liver disease (Montesinos et al. 2002; McBride et al. 2006; Divers 2019; Heatley and Russell 2019; Divers 2020).

The first aim of this study was to establish reference values for BA in plasma from healthy captive green iguanas. The second aim was to determine plasma BA in green iguanas with different types of chronic liver diseases, iguanas suffering from other chronic diseases, and iguanas that have been presented to the clinic for routine or pre-surgical health check-ups.

## MATERIAL AND METHODS

### Animals

The experimental part of the study was performed using a group of 24 healthy green iguanas (*Iguana iguana*) that had been captive-bred and kept for two years under experimental conditions at the Avian and Exotic Animal Clinic, Faculty of Veterinary Medicine, University of Veterinary Sciences Brno. The iguanas were housed in pairs (one male with one female) and maintained in a room with an ambient temperature of 27.9 °C during the day and 24.5 °C at night, general humidity was maintained at 70–80% by daily spraying. Special lamps (Arcadia D3 UV Basking Lamp, 160 W; Arcadia, London, UK) in each enclosure provided a daytime basking area of 35.0 °C and a broad light spectrum (including UVB). The iguanas were exposed to a 12/12 hour light/dark cycle. The diet consisted of dandelion flowers and leaves, lettuce, collard greens, cabbage, and kale, supplemented with calcium powder and commercial iguana pellets (Premium Iguana Adult; Exo Terra, Hagen, Germany). Water was available at all times.

All procedures were evaluated and approved by the University of Veterinary Sciences Brno Animal Care and Use Committee and Committee of Ministry of Agriculture/State Veterinary Administration (VETUNI#11-207/2004; 45450/2019-MZE-18134).

### Blood collection and plasma bile acid determination

The blood samples (0.5–1 ml) were collected using 23Gs 1 Luer needles from the ventral tail vein (*vena coccygea ventralis*), approximately one-third of the tail length caudally of cloaca, to tubes with heparin (Heparin Léčiva inj., Prague, Czech Republic) and centrifuged (5 min, 2 800 *g*, MPW 215; MPW Med Instruments, Warsaw, Poland). Plasma samples were sent to the laboratory and BA concentration was determined either the same day or a few days after storage at –20 °C.

### Samples

The first samples were collected from 24 iguanas at the age of 28 months, and the last samples at the age of 42 months. The range of BA in healthy green iguanas was determined by 5 measurements. To avoid erroneous interpretations of physiologically elevated bile acid concentrations, all green iguanas were fasted for 24 h prior to blood collection. Ten samples with lipemia or haemolysis were not analysed.

The plasma concentrations of BA were measured again in the same group of 18 healthy adult green iguanas (at the age of 42 months) that were fasted for 48 h prior to blood collection.

Furthermore, plasma BA concentrations have been examined in: a) 10 green iguanas suffering from chronic liver disease; b) 10 green iguanas (13 samples) that were suffering from various types of chronic diseases, but without any hepatopathy diagnosed later by histology; c) 18 samples from green iguanas presented to the clinic for routine health check-up; and d) 17 green iguana females with preovulatory follicle stasis (POFS) syndrome presented for health check-up before ovariectomy. All iguanas were regularly fasted for 48 h before the blood collection.

The plasma concentration of bile acids was determined using the enzymatic colorimetric method (Bile Acids; Randox Laboratories, Dublin, Ireland). Shortly, 3α-hydroxy bile acids are converted to corresponding 3-keto-hydroxy bile acids in the presence of NAD^+^ by 3α-hydroxysteroid dehydrogenase. The NADH formed then reacts with nitrotetrazolium blue in a diaphorase-catalysed reaction to form a formazan dye (blue colour with maximum wavelength absorption of 540 nm). The intensity of the colour produced, directly proportional to the bile acids concentration in the sample, is read colorimetrically (Cobas Mira Plus; Roche, Meylan, France).

### Histology

The liver tissue samples collected at necropsy of iguanas that died or patients that were euthanized were fixed in buffered 10% formalin and sent for histological examination to Diagnostic Pathology Laboratory, Dutch Research Institute for Birds and Exotic Animals, The Netherlands or Laboklin GMBH, Germany. Tissue blocks of the fixed liver sample were embedded in paraffin wax, sectioned at 3 μm and routinely stained with haematoxylin and eosin (H&E).

### Ultrasonography

In 3 green iguanas, a 14-year-old male, 4- and 20-year-old females, the control of liver parenchyma was performed as a part of clinical examination with the use ultrasonography (linear probe 4–18 MHz and Ultrasound system RS85; Samsung Medison CO, Seoul, Republic of Korea).

### Statistics

The mean and standard deviation (SD) for plasma BA concentrations were calculated. The significance of the differences between the BA concentrations in plasma samples of healthy green iguanas fasted for 24 h versus 48 h, and groups of healthy iguanas versus iguanas suffering from liver diseases were analysed using the Stat Plus software (v1.01, VUVeL Brno, Czech Republic; Matouskova et al. 1992).

## RESULTS

Mean plasma BA concentration in 110 samples from 24 healthy green iguanas that were fasted for 24 h was 15.89 ± 15.61 μmol/l while the mean BA concentration in 18 samples from the same green iguanas that were fasted for 48 h was (9.56 ± 8.52 μmol/l) (Table 1).

**Table 1:** Plasma bile acids (BA) concentrations in healthy green iguanas

Healthy green iguanas (fasting for 24 h)			Healthy green iguanas (fasting for 48 h)	
Number of plasma samples	BA concentration (mean ± SD)		Number of plasma samples	BA concentration (mean ± SD)
110	15.89 ± 15.61 μmol/l		18	9.56 ± 8.52 μmol/l

**Figure 1:**
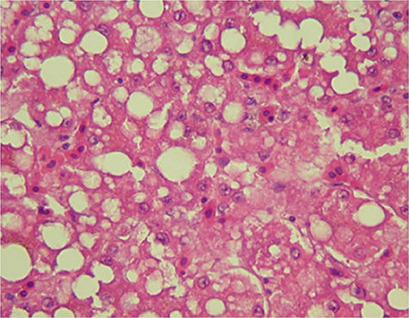
Liver lipidosis (haematoxylin and eosin staining, × 20) *Iguana iguana*

**Figure 2:**
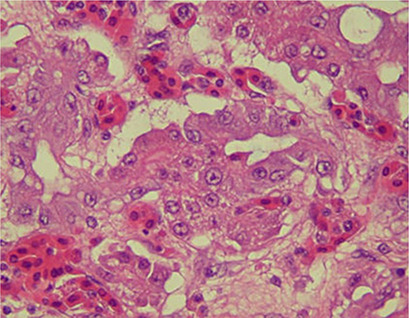
Liver cirrhosis (haematoxylin and eosin staining, × 20) *Iguana iguana*

**Figure 3:**
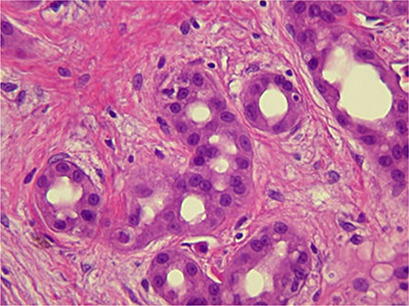
Hepatocellular carcinoma (haematoxylin and eosin staining, × 20) *Iguana iguana*

**Figure 4:**
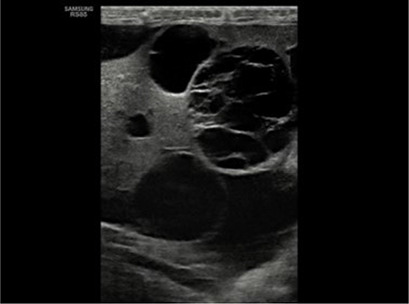
Liver with cysts (Ultrasound system RS85, Samsung Medison CO) *Iguana iguana*

Differences between males and females were not significant (*P* > 0.05). Very high plasma BA concentrations were present in 9 of 10 iguanas suffering from chronic liver diseases (84.85 ± 22.29 μmol/l). In these 9 patients, the BA values were markedly higher (*P* < 0.01) than the BA of healthy iguanas. BA concentrations were significantly altered in 5 patients suffering from liver lipidosis (Figure 1), liver cirrhosis (Figure 2), hepatocellular carcinoma (Figure 3), liver fibrosis, fatty/hydropic degeneration of hepatocytes (diagnoses confirmed by histology); and three patients with hyper or hypoechogenic liver parenchyma with liver cysts (diagnosed by ultrasonography, Figure 4).

BA concentration in one iguana with hepatocellular adenoma (13.0 μmol/l) was within the interval of BA in healthy iguanas (Table 2).

**Table 2:** Plasma bile acids (BA) concentrations in 10 green iguanas suffering from chronic liver diseases

Iguana patients			Type of chronic liver disease	BA concentration (μmol/l)
No.	age (years)	gender		
1	12	M	liver lipidosis	72.03
2	10	F	liver cirrhosis	92.33
3	6	M	hepatocellular carcinoma	120.21
4	9	F	liver fibrosis	104.10
5	17	M	hepatocellular adenoma	13.00
6	12	F	liver fibrosis. generalised hypercalcification	47.20
7	8	F	fatty/hydropic degeneration of hepatocytes	63.50
8	4	F	hyperechogenic liver parenchyma with cysts^a^	82.70
9	14	M	hyper and hypoechogenic liver parenchyma with cysts^a^	100.40
10	20	F	hypoechogenic liver parenchyma with cysts^a^	81.20

Mean ± SD				77.67 ± 32.80
Mean ± SD^b^				84.85 ± 22.29

^a^Diagnosed with the use of ultrasonography; ^b^Nine cases without the case No. 5 (hepatocellular adenoma)

The mean plasma BA concentration in 13 samples of 10 green iguanas that were suffering from various types of chronic diseases, but without any hepatopathy diagnosed by the histology was 7.85 ± 4.86 μmol/l (Table 3).

**Table 3:** Plasma bile acids (BA) concentrations in 10 green iguanas (13 samples) suffering from chronic diseases (without hepatopathies)

Iguana patients			Type of chronic disease	BA concentration (μmol/l)
No.	age (years)	gender		
1	17	M	fibrosing glomerulonephritis	0.50
2	17	F	chronic cystitis, urolithiasis	13.00
3	16	M	renal gout, bacterial pneumonia	7.90
				1.60
4	0.5	M	physical trauma	3.50
5	17	M	myocarditis, pyogranulomatous dermatitis	15.70
6	20	M	sertoli cells tumour	8.40
7	18	M	glomerulonephritis, pneumonia	15.40
8	8	F	glomerulonephritis	4.80
				5.90
				6.20
9	11	M	glomerulonephritis	9.90
10	12	F	myocarditis, pneumonia	10.10

Mean ± SD				7.85 ± 4.86

The mean plasma BA concentration in 18 samples from green iguanas presented to the clinic for routine health check-ups was 11.95 ± 9.43 μmol/l (Table 4).

**Table 4:** Plasma bile acid (BA) concentrations in 18 green iguanas presented for health check-up

Iguana patients			The purpose for plasma chemistry	BA concentration (μmol/l)
No.	age (years)	gender		
1	16	M	health check-up	8.20
2	17	M	health check-up	9.30
3	12	F	health check-up before surgery	2.10
4	8	M	health check-up before surgery	12.60
5	8	F	health check-up	17.50
6	3	F	health check-up	0.50
7	4	M	health check-up	10.00
8	7	F	health check-up before surgery	30.70
9	9	M	health check-up before surgery^a^	34.50
10	9	F	health check-up before surgery	14.60
11	12	F	health check-up before surgery	18.50
12	18	F	health check-up before surgery	0.90
13	8	M	health check-up	9.90
14	11	F	health check-up before surgery	16.20
15	19	M	health check-up before surgery	3.30
16	23	M	health check-up, problems with locomotion	14.10
17	16	F	health check-up	2.50
18	10	M	health check-up before surgery	9.70

Mean ± SD				11.95 ± 9.43

^a^Ascites

Plasma bile acid (BA) concentrations in 17 green iguana females with preovulatory follicle stasis (POFS) syndrome presented for ovariectomy was 12.97 ± 9.06 μmol/l (Table 5).

**Table 5:** Plasma bile acids (BA) concentrations in 17 green iguana females with POFS syndrome

Iguana females		BA concentration (μmol/l)
No.	age (years)	
1	2	4.60
2	2	18.90
3	2.5	5.60
4	2	10.90
5	2.5	28.00
6	2.5	4.90
7	2	11.80
8	8	9.80
9	2	1.40
10	5.5	21.60
11	2	14.20
12	4	10.30
13	3	2.10
14	3.5	9.10
15	10	20.60
16	9	33.90
17	3.5	12.80

Mean ± SD		12.97 ± 9.06

POFS = preovulatory follicle stasis syndrome

## DISCUSSION

The mean plasma bile acids concentration in 110 samples from 24 healthy green iguanas (fasted for 24 h) was higher than BA observed in a previous study (McBride et al. 2006). In 18 samples from the same healthy green iguanas (fasted for 48 h), the mean plasma bile acid concentration (9.56 ± 8.52 μmol/l) was very similar to the results of McBride et al. (2006). The data collected from the pilot study suggested that 3α-hydroxy bile acid concentrations would be significantly increased by feeding for at least 8 h (McBride et al. 2006), and these authors recommended to fast green iguanas for 48 h prior to bile acid determination (Divers 2020). This is in accordance with our current results. The broad spectrum of variance (mean ± SD) for bile acid concentrations has been observed in both studies. It remains open whether the pressure exerted externally during restraint may result in contraction of the gall bladder thus altering the plasma bile acid concentration (McBride et al. 2006). All iguanas in the present study were handled gently by a skilled technician. Bile acids are commonly assayed and used in some reptiles as an indicator of hepatic dysfunction (Heatley and Russell 2019). Bile acids are derived from cholesterol and synthesised in the liver (Divers 2019).

In reptiles with chronic liver diseases, enterohepatic cycling and efficiency of ileal reabsorption could be negatively influenced and the efficiency of liver parenchyma for reabsorption and metabolic reconstruction of bile acids decreased. Such a situation may result in an altered concentration of bile acids in peripheral blood. While in red-eared terrapins a postprandial increase in bile acids was not seen (Knotkova et al. 2008) and the correlation of bile acid concentrations with histopathologic changes in the liver has not been described for chelonians (Innis and Knotek 2020), in lizards (green iguanas and bearded dragons) the assay was used successfully for evaluation of postprandial bile acid increase in fasting lizards (Divers 2020).

The mean plasma BA concentration in green iguanas suffering from various types of chronic diseases, but without any hepatopathy diagnosed by histology or ultrasonography was low and within the range of BA concentration for healthy green iguanas. BA concentration in green iguanas presented to the clinic for routine health check-ups as well as in female green iguanas with POFS syndrome presented to the clinic for ovariectomy was within the interval for BA in healthy green iguanas.

The data collected from this study suggest that 3α-hydroxy plasma bile acids are significantly increased in green iguanas suffering from chronic liver diseases. The highest plasma BA concentrations were present in iguanas suffering from liver lipidosis, liver cirrhosis, hepatocellular carcinoma, liver fibrosis, fatty/hydropic degeneration of hepatocytes, and patients with hyper- or hypoechogenic liver parenchyma with cysts. The BA concentration, however, in one iguana with hepatocellular adenoma was within the interval of BA in healthy iguanas (13.0 μmol/l). The feasibility of bile acids concentration assay has been documented in just a few reptile species up to date and more research is needed for further progress on this field.
